# Postoperative outcome analysis of left-sided idiopathic diaphragmatic hernia: a case report

**DOI:** 10.11604/pamj.2022.43.113.35652

**Published:** 2022-10-31

**Authors:** Anam Rajendra Sasun, Vishnu Diwakar Vardhan, Samiksha Vinod Sonone

**Affiliations:** 1 Ravi Nair Physiotherapy College, Datta Meghe Institute of Medical Sciences, Sawangi Meghe, Wardha, Maharashtra, India

**Keywords:** Idiopathic diaphragmatic hernia, laparotomy repair, physiotherapy interventions, tele-rehabilitation, case report

## Abstract

Diaphragmatic hernia of an idiopathic cause is a rare pathology that occurs due to absence of any trauma or congenital cause. The aim of the study was to report a case of left-sided diaphragmatic hernia without traumatic aetiology. A 59-year-old male had complained of epigastric pain, vomiting, nausea, and breathing difficulties for the past 15 days. After investigations and diagnosis of the condition, the patient subsequently underwent laparotomy surgery to repair the defect. Outcome measures like FIM, NPRS, Incentive Spirometry, ICU-MS, CPaX, and HADS were used to assess the recovery of the patient. Surgical management of the condition was achieved, but to bring the patient back to his pre-pathology life without signs of breathlessness, and fatigue, a comprehensively designed physiotherapy rehabilitation is very beneficial. Our case report is the first-ever report made on physiotherapy management of diaphragmatic hernia after surgery. Tele-rehabilitation had been absolutely vital in patient follow-up.

## Introduction

Diaphragmatic hernia is a lesion in the diaphragm in which loops of the small and large bowel, stomach, liver, and spleen can bulge into the thoracic cavity of the involved side. This defect can be classified as congenital or acquired [1]. Congenital diaphragmatic defects allow the abdomen´s visceral contents to pass into the thoracic cavity. These hernias are classified into three types, Morgagni hernia, Bochdalek hernia, and Hiatal hernia. The delayed presentation has been observed in 2.5-20% of all patients [2]. Bochdalek hernia is an extremely rare lethal condition, causing incarceration and strangulation, accounting for 31% of the mortality rate. Morgagni hernia is described as herniation of abdominal content through an anterior defect. Its features include nonspecific respiratory and gastrointestinal symptoms [3].

Idiopathic diaphragmatic hernia is a rare pathology with various respiratory and gastrointestinal symptoms [4]. The surgical repair of the defect is done through Laparotomy, Thoracotomy, or both. Laparoscopic repair has become possible with the recent advancement of Minimally Invasive Surgery [5]. The site and length of the incision, anaesthetic effect, reduced mobility, all of these factors collectively have a huge impact on respiratory muscle function and diaphragmatic movements. This results in suppression of cough reflex followed by secretion retention and reduced lung volumes, all of which collectively result in lung atelectasis and infection [6]. We hereby report a case of adult-onset idiopathic diaphragmatic hernia repaired with laparoscopy surgery and the crucial role of physiotherapy in bringing back the patient to his daily living. Thereby, improving quality of life.

## Patient and observation

**Patient information:** a 59-year-old male patient, alcoholic since 20 years, Tobacco chewer since 25 years, non-smoker with medical history of grade (II) prostomegaly for the last 06 months. Patient presented with complaints of pain in the left upper abdomen, nausea, vomiting, and breathlessness for the past 15 days.

**Clinical findings:** he described his abdominal pain to be insidious in onset, precipitating factors being walking and work while relieving factors for pain being consumption of proton pump inhibiter (Pantoprazole). No h/o abdominal trauma and any congenital deformity. On general examination, Vitals were: Blood pressure 128/80 mmHg. Pulse rate 88 beats/min, and Oxygen saturation of 97%. The examination was done after taking properly written and explained consent from the patient. The examination was done on POD day 2. On inspection and observation, the patient was examined in the propped-up position with an ample amount of support through pillows at the back. On inspection, the patient was on 4L of face mask Oxygen support followed by a Central line, Foley´s catheter, Ryle's tube, ICD, and left pelvic drain (in-situ). Chest movements were reduced, thoraco-abdominal type with a respiratory rate of 19 Breaths/min. All findings were confirmed on palpation. Chest expansion at the axillary nipple, and xiphisternum was done with differences of 2cm, 2cm, and 1cm. Tenderness is present in an epigastric area. Auscultation revealed, a reduction in air entry bilaterally upon lung parameters. The patient demonstrated ICU-acquired weakness.

**Figure 1:**
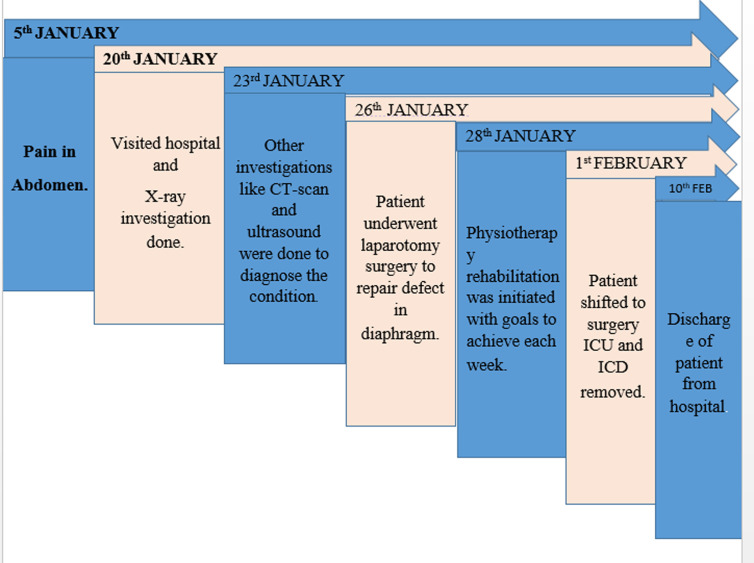
describing timeline of events

**Timeline of the current episode:** the entire timeline of events is illustrated in (Figure 1).

**Figure 2:**
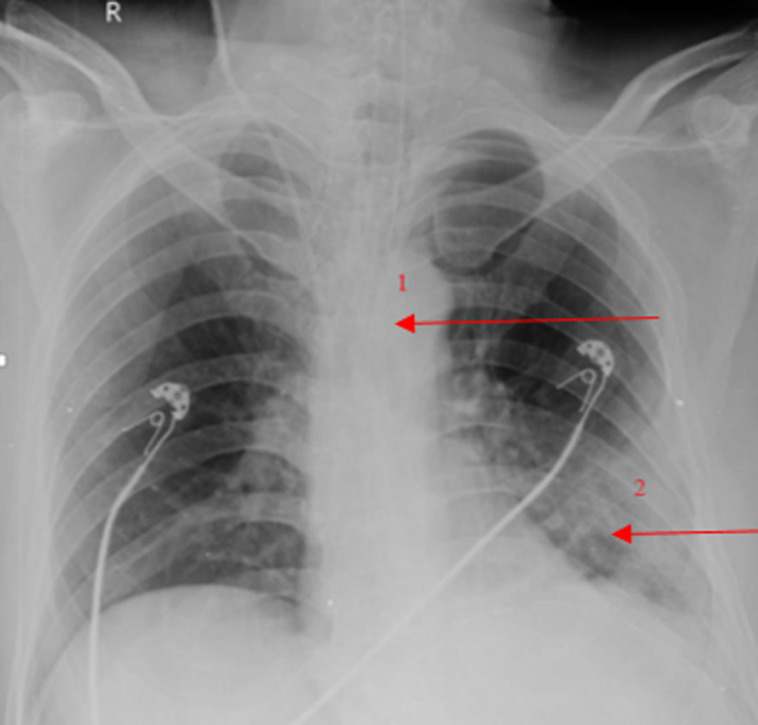
chest x-ray with right mediastinum shift and collapse of left basal lung

**Figure 3:**
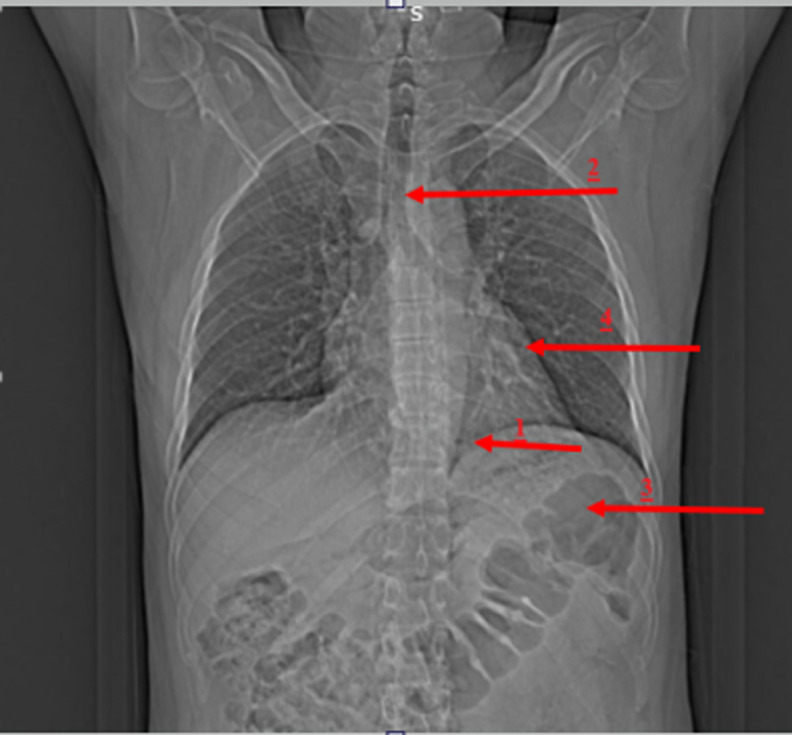
computed tomography showing invasion of abdominal contents into thoracic cavity due to herniation

**Diagnostic assessment:** X-ray chest showed elevation of the left dome of the diaphragm with a mediastinum shift towards the right side, with collapse of the left basal lung (Figure 2). CT Impression of Abdomen and Pelvis showed large defect in left crus of the diaphragm with herniation of stomach to the left thoracic cavity, along-with shift of mediastinum towards the right side (Figure 3). USG impression of Abdomen Pelvis: Liver, Gall bladder, Pancreas, Kidney, Spleen, and bladder appeared normal in morphology and echo-texture. Prostate: Size 4.8×4.3×3.9 cms, weight 46gms, volume 44.1 ml. The enlarged, median lobe of the prostate is protruding into the lumen of the urinary bladder. Laboratory Investigations revealed some derangements (Table 1). Due to financial issues, other investigations couldn't be performed.

**Table 1:** post-operatively baseline derangement

Parameter	Result	Unit	Value
Haemoglobin	11	gm%	12-18
White blood	3800	/cmm	4000-11000
Red blood count	3.70	mil/cmm	4.5-6.5
Haematocrit	34.9	%	36-54
Neutrophils	80	%	50-65
RDW-CV	16.2	%	36-54
Calcium	8.2	mmol/L	8.4-10.2
Chloride	108	mmol/L	98-107
KFT (Na+)	132	mmol/l	136-145
KFT (K+)	4.3	mmol/L	3.5-5.1
Phosphorus	4.6	mmol/L	2.5-4.5
Bicarbonates	21	mmol/L	25-29
COVID-19 IgG, IgM	Negative		

RDW-CV: Red Cell Distribution Width: Coefficient Variant. KFT: Kidney Function Test. COVID-19: Corona Virus Disease 19. IgG, IgM: Immunoglobulin G and Immunoglobulin M.

**Diagnosis:** the diagnosis “idiopathic diaphragmatic hernia” of left side was confirmed on the basis of various diagnostic assessments and investigations.

**Figure 4:**
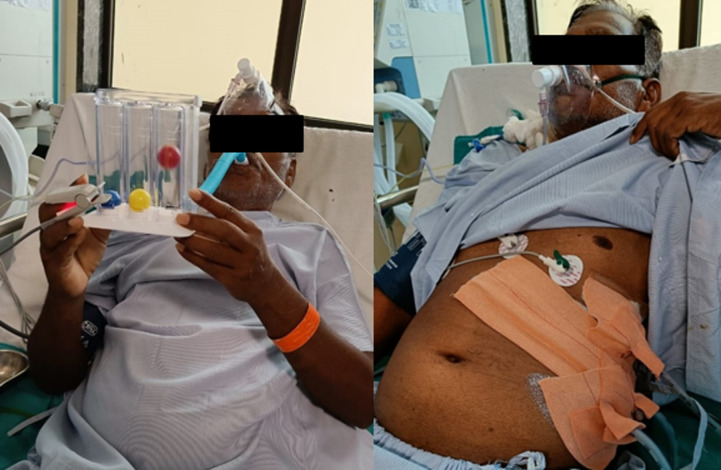
patient performing incentive spirometry of 6oo cc with rest intervals

**Therapeutic interventions:** patient is managed medically with antibiotics, antacids, and multivitamins. The major intent of our designed physiotherapeutic rehabilitation was to improve his quality of life and allow him to return to his daily activities with as little fatigue and shortness of breath as possible. (Figure 4). Physiotherapy rehabilitation was given for 2 weeks followed by a regular follow-up (Table 2). The whole of the week 1 protocol was followed along with some additional interventions for Week 2 described in (Table 3).

**Table 2:** describing various physiotherapeutic interventions for week 1 rehabilitation

SR. No	Physiotherapy goals	Therapeutic intervention	Treatment regimen
1.	To reduce pain at suture site	Cryotherapy	Cryotherapy was applied wrapped in a towel for 03 minutes twice a day
2.	To fasten the recovery, wound healing, and reduce strain over suture incision	Abdominal binder	Supports during mobility
3.	To encourage airway clearance	Chest percussions and vibrations	1. Initial 2 days
			2. 4-8 days
		Manual Assisted huffing and coughing	
4.	To educate the patient and family members about his current health status	Patients and family members should be educated about the importance of designed rehabilitation and exercise regimens	Education about early ambulation, positioning, and resuming Activities of daily living
5.	To avoid post-surgery integumentary, circulatory, and pulmonary complications	1. Initially, the patient was positioned in a semi-fowlers position	1. Initially, positioning was given every 2 hours
			2. 20 reps× 1 set twice a day, later 30 reps×2 set 3-4 times a day
		Later bedside sitting was given	
		2. Ankle Toe movements	
6.	To improve IRV and FRC of lungs	1. Thoracic Expansion exercises with 180-degree shoulder flexion and 60 shoulders extension followed by deep expiration at the end initiated	1. 10 reps×1set twice a day
			2. Started from POD-2, initially 3-4 times a day with rest bouts
			Lately, the patient was instructed to perform every 2 hours
		2. Incentive spirometry started from POD 3 and feedback was obtained visually through different coloured balls of red, yellow, and blue representing 600, 900, and 1200 c.c each	
7.	To reverse ICU acquired weakness and prevent joint stiffness	Initially Active Range of Motion exercises to U/L and L/L. Later, progressed with mild to moderate resistance application	For initial 5 days, 15 reps× 1 set twice a day. Later, 15 reps× 2 sets thrice a day
8.	Improve breathing patterns and respiratory rate	Deep breathing exercises like 360-degree breathing, Diaphragmatic breathing, and segmental breathing were taught	15 reps× 2 sets thrice a day were taught with a rest interval of 30 sec between each set
9.	To prevent Deep Vein Thrombosis	Crepe-bandages	The bandage was applied in the figure of eight patterns in both LL

Reps: Repetitions U/L and L/L: Upper and lower limb POD: Post-operative day. ICU: Intensive Care unit

**Table 3:** describing various physiotherapeutic interventions for Week 2 rehabilitation

SR. No	Physiotherapy goals	Therapeutic intervention	Treatment regimen
1.	To encourage airway clearance.	Autogenic drainage, active cycle of breathing exercises.	9^th^ day onwards.
2.	To improve the strength of respiratory muscles.	Using a device named inspiratory muscle trainer.	Starting on day 8^th^, the patient was instructed to perform with rest intervals.
3.	To increase strength, endurance, and power of U/L and L/L muscles and reverse ICU acquired weakness.	Progression was done with thera band strengthening followed by weight cuff training starting initially with 500mg with progression to 1 kg.	15 reps×1 set twice a day with regular rest intervals.

U/L: Upper limb. L/L: Lower limb. Reps: Repetitions. ICU: Intensive care unit

**Figure 5:**
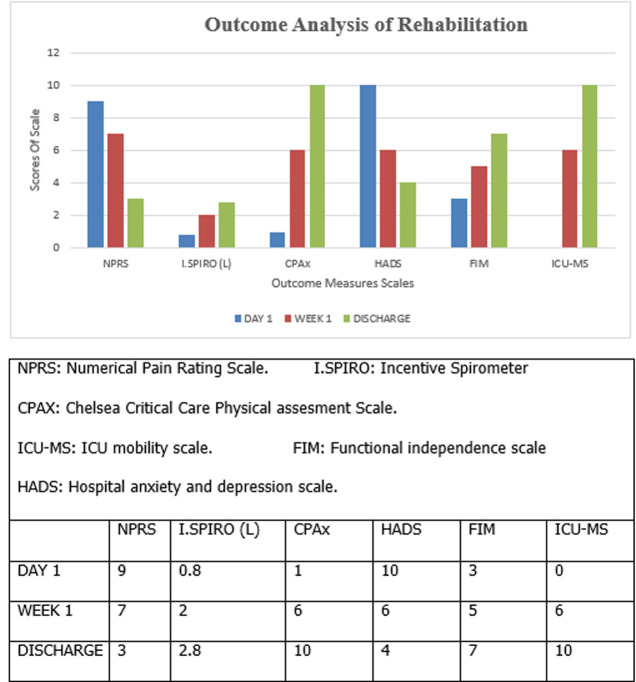
describing outcome analysis of rehabilitation

**Follow-up and outcome of interventions:** the patient showed outstanding response our rehabilitation when compared with Day 1, Week 1, and Day of Discharge status. Follow-up was done every 15 days (Figure 5) (Table 4).

**Table 4:** outcome analysis of different scales used for tracking the progress of the patient

Scales	Day 1	Week 1	Discharge
NPRS	9	7	3
INC.SPIRO (L)	0.8	2	2.8
ICU-MS	0	6	10
FIM	3	5	7
CPaX	3	4	5
HADS	10	6	4

ICU-MS: ICU Mobility Scale. CPAx: Chelsea Critical Care Physical Assessment Tool. HADS: Hospital Anxiety and Depression. FIM: Functional Independence Measure

**Patient perspective:** “I feel blessed to have undergone physiotherapy rehabilitation after my surgery, I would like to thank my therapist with helping me to return back to my daily living. I assure you to continue with the treatment and regular follow-ups”.

**Informed consent:** A well-explained and oral consent was obtained from the patient. The patient was very happy with the various physiotherapeutic interventions given and the progression in his health status and was highly satisfied with his progress.

## Discussion

The history of Diaphragmatic hernia starts with Lazarus Riverius, who discovered this entity during a post-mortem study of a 24-year-male. Idiopathic Diaphragmatic hernia occurs, with no obvious cause. Left-sided predisposition is due to the liver's protection and in part to the fact that embryonic fusion defects are more common in the left. This spontaneous form may be asymptomatic for decades before becoming symptomatic in the late stage when it has expanded [7]. Use of spirometry was found very effective in preventing postoperative complications following abdominal surgery. The postoperative complications can be hypoxemia, early fatigue, decreased inspiratory muscle strength, and atelectasis. He even concluded that 6MWT, volume-incentive spirometry, and flow-metric spirometer showed statistically greater improvement at the time of discharge, which is the results of our treatment [8].

Another added benefit of physiotherapy is that it can support bowel control, notably when coughing or having good expiratory movements. Because of prolonged immobility and resting periods, patients show negative impacts, therefore early ambulation is a must. The insight into the extended ramification of ICU survivorship has fuelled interest in rehabilitation. ICU acquired weakness occurs within the first 48 hours, so it is crucial to limit muscle weakness and post-intensive care syndrome. Physiotherapists are prominent figures of the inter-professional ICU team because they are skilled in multi-system assessment and management of intubated and spontaneously breathing patients. Active mobilization followed by physiotherapy treatment increases Muscle Strength, and Functional Independence, if initiated within the starting days of ICU admission [9]. Physiotherapy involving lung fields have generally been a prominent regimen of post-operative treatment following abdominal surgery, to limit or minimize problems such as lung atelectasis, pneumonia, sputum retainment, and restrictive lung pathology with alterations in pulmonary structures [10]. Implementing the Autogenic Drainage technique into routine chest physiotherapy after upper abdominal surgery improves blood gases exchanges, shortens hospital stay, and is associated with a lower percentage of pulmonary complications. Furthermore, patients tolerate it very well. As a result, removing infected secretions from the airways can increase ventilator capacity, thereby decreasing direct inflammation of the airway cells [11].

This study is remarkable as such pathologies require surgical emergencies, accounting for less than 1% of all diaphragmatic hernias and with fewer than 30 reported cases in the literature [12]. Our case report is the first-ever report made on novel physiotherapeutic rehabilitation for laparoscopic repair of left-sided idiopathic diaphragmatic hernia. The report has described detailed rationale, interventions, and treatment regime of various interventions which have helped our patient to resume back his daily life and improve his health-related quality of life.

## Conclusion

Our rehabilitation facilitated the patient's recovery by avoiding postoperative complications of laparoscopic diaphragmatic hernia and by implementing various physiotherapeutic approaches and making patients return to their pre-pathological status. A 2 week-physiotherapy regime had a good and beneficial impact on the patient and subsequently allowed him to start his ADLs at the time of discharge patient's full recovery was not achieved, but a major percentage of therapeutic objectives were met, including improved chest expansion, breathing pattern, increased functional vital capacity of lungs, pain reduction, and resuming daily activities after 2 weeks of intensive physiotherapy rehabilitation. The patient was instructed to come for a follow-up every 15 days.
